# Finding truth in an ocean of evidence

**DOI:** 10.1093/hropen/hoac004

**Published:** 2022-02-09

**Authors:** Siladitya Bhattacharya, Edgardo Somigliana, Andrew Williams

**Affiliations:** 1 Editor-in-Chief, Human Reproduction Open; 2 Deputy Editor, Human Reproduction Open; 3 Managing Editor, ESHRE Journals

## Published evidence: the current situation

Over 1 million biomedical publications are added to PubMed each year. Meanwhile, the internet offers the lay public a seemingly endless quantity of scientific information, opinions and beliefs. The sheer volume of information available presents a major challenge to clinicians who are expected to make everyday decisions based on the best available evidence. Many exciting discoveries in our field come to our notice through uncontrolled or poorly controlled observational studies and need robust evaluation in the context of randomized trials before they can be adopted into routine clinical practice. This process takes time and, in some cases, may not happen at all. Clinicians face a quandary—do they accept results based on suboptimal study designs or risk rejecting potentially effective treatments in their quest for epistemic certainty. Part of the challenge in identifying evidence we can trust is the proliferation of low-quality and misleading publications which can obscure the truth. How can researchers, journals, clinicians and patients play a part in improving this interface between science and health care delivery?

## The nature of scientific enquiry in medicine

Science is, and has always been, corrigible—with most advances occurring in incremental steps. Acceptance of error is central to a scientific approach where hypotheses are tested and, if proved wrong, rejected. Willingness to live with uncertainty is a key attribute of science and a strength rather than a weakness in those who believe in scientific thought. As this legitimate element of uncertainty should not be allowed to paralyse clinical decision-making, we need a dynamic process of decision-making using evidence available at any given time, to guide treatments which are effective, safe and acceptable.

Acceptance of evidence-based medicine implies awareness of the variable quality of evidence within the scientific literature. Researchers know that the chances of error can be minimized by choosing the most appropriate study design, sample size and outcomes for a study and by actively taking steps to minimize bias. Better understanding of tests of statistical significance and conscious steps to avoid ‘p hacking’ have also led to better interpretation of quantitative data. At the same time, the appetite for positive novel findings carries predictable risks of publication bias and poor replicability.

Even well-designed and properly conducted research is not free from random errors. Randomized trials are generally assumed to be the best way of evaluating clinical interventions. Yet, even in well-designed trials comparing two treatments with 80% power and a 5% level of significance, 1 in 5 studies will fail to detect a true difference between 2 interventions, while 1 in 20 will falsely identify a difference where none exists. Publishing findings other than those identified *a priori* as either primary or secondary outcomes can limit the risk of populating the literature with misleading results.

A striking development in scientific research over the past few decades has been the growth of secondary research in the form of systematic reviews and meta-analyses. Meta-analysis is a powerful tool which allows us to aggregate the results of well-designed but underpowered studies to provide a definitive answer to clinical questions and are often used to inform clinical practice guidelines. At the same time, indiscriminate aggregation of data without careful clinical or methodological considerations can seriously compromise the quality of the output. This can be worrying enough in the context of randomized data with significant clinical or statistical heterogeneity but is much worse in the case of observational studies, where meta-analyses of crude unadjusted results can generate greater degrees of precision around potentially erroneous results. In the absence of the most appropriate research, it is not unreasonable to explore all available data—but of course this needs a more nuanced appraisal of the margin of effect, risks, costs and acceptability associated with alternative interventions.

## The role of scientific journals

Scientific journals have a clear responsibility in terms of curating high-quality scientific output and making it accessible to consumers. It goes without saying that published research should be genuine and ethical, and journals need to be more proactive in ensuring the provenance of what they publish. Data should be presented in a transparent way and all conclusions drawn from them should be valid. As scientists are just as likely to be wedded to certain preconceptions or beliefs as other humans, it is important that published papers are clear about any confirmation bias. Overstatement of the clinical relevance of the findings is a common fault among researchers which needs to be moderated by peer reviewers. Journals also have a key role in combating publication bias by demonstrating their willingness to publish papers reporting negative results.

Through a robust system of supportive peer review, journals need to make sure that readers get a clear sense of the veracity, precision and limitations of published research. While true for all scientific journals, this is especially important for all medical journals which mainly report on clinical evidence which can directly impact on patient care.

Journals also have a responsibility in educating readers to become sophisticated consumers by giving them the tools to critically appraise published material and by ensuring that all output is freely accessible. Finally, for maximum impact, medical journals should try to communicate effectively with a mixed audience of scientific and non-scientific readers.

Evidence-based medicine relies on our ability to critically evaluate research findings to inform clinical practice. This is facilitated by open access journals like *HROpen* which make scientific findings freely accessible to all, and use a rigorous peer review process to ensure that all evidence is presented in a transparent, unbiased and comprehensible manner.

